# Learning curve evaluation upskilling retinal imaging using smartphones

**DOI:** 10.1038/s41598-021-92232-w

**Published:** 2021-06-16

**Authors:** Linus G. Jansen, Payal Shah, Bettina Wabbels, Frank G. Holz, Robert P. Finger, Maximilian W. M. Wintergerst

**Affiliations:** 1 Department of Ophthalmology, University Hospital Bonn, Venusberg-Campus 1, 53127 Bonn, Germany; 2 Sankara Academy of Vision, Sankara Eye Hospital Bangalore, Varthur Main Road Kundalahalli Gate, Bangalore, 560037 India

**Keywords:** Medical research, Translational research, Biomedical engineering, Eye diseases

## Abstract

Smartphone-based fundus imaging (SBFI) is a low-cost approach for screening of various ophthalmic diseases and particularly suited to resource limited settings. Thus, we assessed how best to upskill alternative healthcare cadres in SBFI and whether quality of obtained images is comparable to ophthalmologists. Ophthalmic assistants and ophthalmologists received a standardized training to SBFI (Heine iC2 combined with an iPhone 6) and 10 training examinations for capturing central retinal images. Examination time, total number of images, image alignment, usable field-of-view, and image quality (sharpness/focus, reflex artifacts, contrast/illumination) were analyzed. Thirty examiners (14 ophthalmic assistants and 16 ophthalmologists) and 14 volunteer test subjects were included. Mean examination time (1st and 10th training, respectively: 2.17 ± 1.54 and 0.56 ± 0.51 min, *p* < .0001), usable field-of-view (92 ± 16% and 98 ± 6.0%, *p* = .003) and image quality in terms of sharpness/focus (*p* = .002) improved by the training. Examination time was significantly shorter for ophthalmologists compared to ophthalmic assistants (10th training: 0.35 ± 0.21 and 0.79 ± 0.65 min, *p* = .011), but there was no significant difference in usable field-of-view and image quality. This study demonstrates the high learnability of SBFI with a relatively short training and mostly comparable results across healthcare cadres. The results will aid implementing and planning further SBFI field studies.

## Introduction

Smartphone-based fundus imaging (SBFI) takes an increasingly important role for screening purposes in a variety of diseases, and has been shown to represent an alternative to conventional imaging in different settings^1–14^. It is of special interest in resource limited settings^1–6^. In low- and middle-income settings, it has the potential to increase the availability of eye care, and thus, prevent avoidable visual impairment^15,16^. The relative scarcity of ophthalmologists in many low- and middle-income countries necessitates trained ophthalmic assistants for screening purposes, which has also been emphasized by the World Health Organization and the International Diabetes Federation in terms of enhancing the effectiveness and efficiency of care delivery by task shifting and delegation^17–21^. However, to date there is a dearth in the literature on the time needed to sufficiently train for SBFI and it remains unclear whether non-expert operators are able to achieve results comparable to experts. Evidence from several studies suggests non-expert examiners can learn and employ SBFI with sufficient results^22–29^. Queiroz et al. evaluated the learning curve of nurses carrying out SBFI and concluded that 80% of the acquired images were usable for clinical decisions^41^. However, other studies reported inability to detect diabetic retinopathy employing SBFI by non-expert examiners^30^. The reason for these observed differences is unclear, to date.

To fill this gap, we compared the learning curve of ophthalmic assistants and ophthalmologists in SBFI in terms of examination time, total number of images, image alignment, usable field-of-view, and image quality for a novel SBFI device.

## Methods

### Setting and participants

Ophthalmic assistants and ophthalmologists without previous experience in SBFI were prospectively included in the study as examiners. Volunteers were included as subjects for examination. Ethical approval was obtained from the human research ethics committee of the University of Bonn, Germany (ethics approval ID 209/16) and informed consent was obtained from both the examiners and the volunteers. Volunteers had one eye dilated with tropicamide (5.0 mg/ml) and phenylephrine (100 mg/ml) and were seated, imaging took place at least 30 min after initiation of dilation. Sufficiency of pupil dilation was verified before initiation of the examination and if needed further dilating eye drops were applied. Examiners were either sitting or standing according to their preference. The examination was carried out in a darkened room. Ophthalmologists included were either residents or consultants. Ophthalmic assistants recruited were doing their optometry graduation or had just completed their course and started working.

### Device

**Figure 1:**
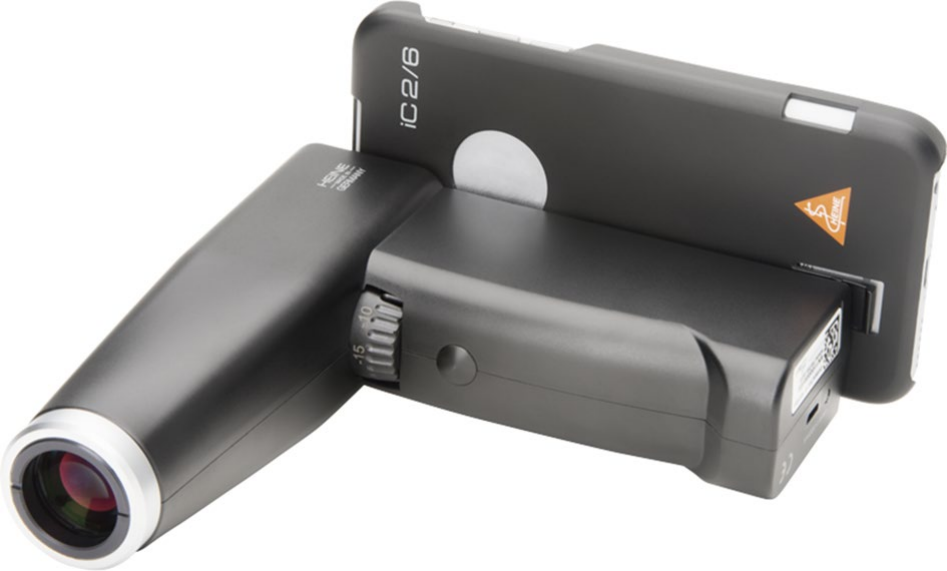
The smartphone-based fundus imaging device used in this study. Source:^31^.

An iPhone 6 (Apple Inc., Cupertino, California, USA) with the Heine iC2 (see Fig. 1) as a SBFI adapter were used in this study^31^. The SBFI adapter was unknown to all participants and was connected to the smartphone via Bluetooth and an application developed by the manufacturer of the device was installed on the mobile device in order to connect and operate the SBFI adapter. The SBFI adapter has a trigger for image acquisition with two pressure points similar to a standard digital single-lens reflex camera. Alternatively, image acquisition was performed by a button on the smartphone’s touch screen. The device requires pupil dilation and the maximum achievable field-of-view of the device is 34°. Refraction was adjusted by a diopter wheel while visually controlling the sharpness/focus of the image. The light source is part of the adapter, its brightness was adjusted for optimum illumination and patient comfort. Single-image mode was used for image acquisition.

### Standardized introduction to the device

A short standardized three minute introduction regarding the handling of the device was given to the participants before initiating the first examination. The introduction was carried out in person by one of two instructors experienced in SBFI (PS or LGJ) and included a basic explanation on how to use the device and the application installed on the smartphone. Additionally, technical questions were answered. As the intention of the study was to investigate how SBFI can be learnt through ‘learning by doing’, which is often the case in real-world clinical settings, we minimized the theoretical input during the training and no image acquisition was done during the introduction. Therefore, the first images captured directly after the training without any prior practice were already part of the data collection.

### Examination and data acquisition

Each participant carried out 10 examinations (‘training cycles’) consecutively. The aim of each examination cycle was to capture two images of the central retina: one centered on the optic disc and one centered on the macula whilst trying to achieve the best possible image quality in terms of sharpness/focus, reflex artifacts and contrast/illumination. Correct alignment of the optical path was achieved by providing external fixation reference points (e.g. a landmark in the examination room) to the participants and by properly positioning the device. Participants were allowed to take multiple images of each location. After each examination cycle, the examiner manually selected the best images to be saved for analyses and the remaining images were discarded. The time needed for each examination was documented. Examination time started after the patient was seated and the examiner was in position, and before the examiner positioned the device. Examination time ended when the examiner finished image acquisition.

### Image and statistical analyses

For each examination cycle the time needed for image acquisition, and the total number of images were analyzed. Up to 5 images per single location (macula or optic disc centered) were included in the analyses. All images were graded for image alignment (deviation from the optimal alignment in pixels), usable field-of-view (in percent), and image quality in terms of sharpness/focus, reflex artifacts, and contrast/illumination. Image quality was graded using the semi-quantitative scales established by Wintergerst et al.^6^ Image alignment was assessed by measuring the distance between the position of the optimal and actual image center in pixels using Fiji^32^ (an expanded version of ImageJ^33^) and the field-of-view was estimated by subjective evaluation. All analyses were performed masked. The Kruskal–Wallis/Wilcoxon test was used for independent/repeated multiple comparison between groups for non-parametric data and ANOVA for parametric data. Post-hoc analysis for examination time was performed using Pairwise Wilcoxon Rank Sum Test. Statistical analyses were performed with R (R: A Language and Environment for Statistical Computing, R Core Team, R Foundation for Statistical Computing, Vienna, Austria, version 4.0.3) and figures were produced using the package ggplot2 (Wickham H 2016. ggplot2: Elegant Graphics for Data Analysis. Springer—New York. ISBN 978–3-319–24,277-4, https://ggplot2.tidyverse.org). All methods were carried out in accordance with relevant guidelines and regulations.

## Results

### Demographics

Thirty examiners (14 ophthalmic assistants and 16 ophthalmologists, mean age 27.27 ± 4.11, age range 20 – 38, 60% female) were included in the study. All of the 14 volunteer test subjects (mean age 23.23 ± 3.45, age range 18 – 32, 67% female) were phakic. None of the ophthalmic assistants had previous experience in ophthalmoscopy.

### Effect of training on examination time

**Figure 2:**
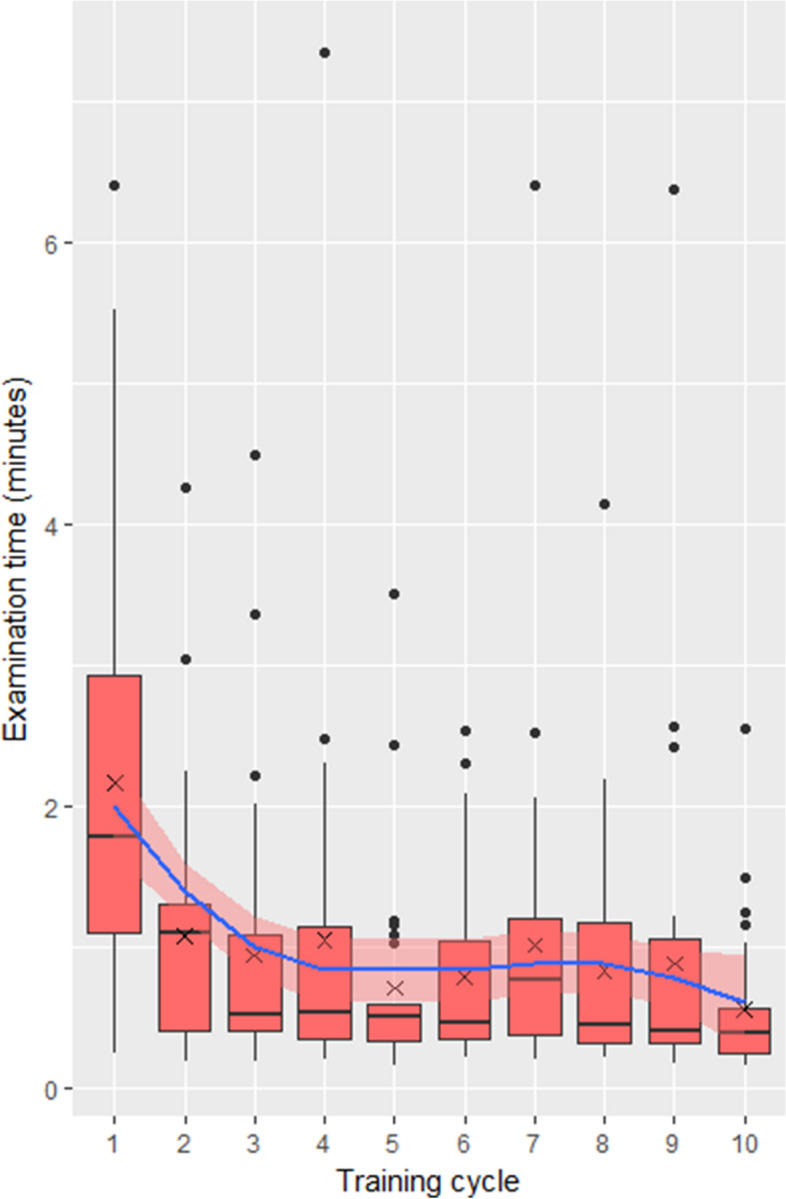
Effect of training on examination time. Boxplot values over 1.5 interquartile range below the first quartile or above the third quartile were defined as outliers. Crosses indicate the mean. The blue line indicates a local polynomial regression fitting with 95% confidence intervals in light red.

Mean examination time of the tenth cycle was significantly shorter than the first (0.56 ± 0.51 and 2.17 ± 1.54 min, respectively, Wilcoxon signed-rank test *p* < 0.0001) with a mean improvement in examination time of 1.61 ± 1.63 min (see Fig. 2). Examination time significantly correlated with the number of training cycles (Spearman correlation coefficient r = -0.31, *p* < 0.0001). The total time needed for the complete training including the initial instruction and the 10 training cycles was about 30 min.

### Effect of training on usable field-of-view and image alignment

The mean usable field-of-view of the tenth cycle was significantly larger compared to the first cycle (92 ± 16% and 98 ± 6.0%, respectively, Wilcoxon signed-rank test *p* = 0.003). Percent usable field-of-view significantly correlated with the number of training cycles (Spearman correlation coefficient r = 0.080, *p* = 0.029). Image alignment was not significantly different between the first and tenth cycle (202 ± 113 pixels and 168 ± 90 pixels, Wilcoxon signed-rank test *p* = 0.087).

### Effect of training on image quality

**Figure 3:**
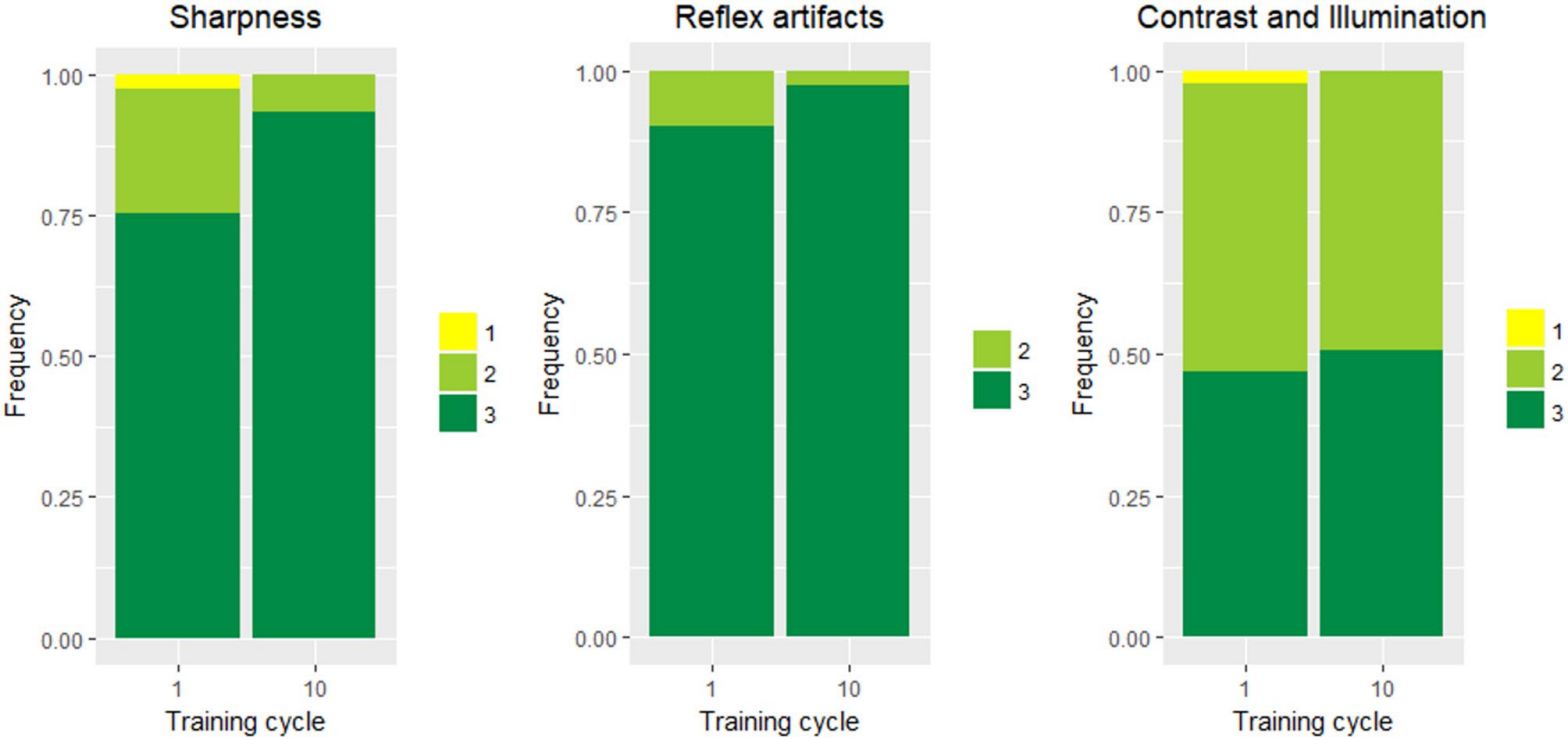
Effect of training on image quality in terms of sharpness/focus, reflex artifacts and contrast/illumination. The frequencies of image quality grades for sharpness/focus (**left**), reflex artifacts (**middle**) and contrast/illumination (**right**) are displayed for the first and last training cycle. Higher grades correspond to a better image quality (see Wintergerst MWM et al. 2020^6^ for the respective semi-quantitative image quality scales which have been used for analysis).

Image quality improved by training in terms of sharpness/focus, but not in terms of reflex artifacts and contrast/illumination (see Fig. 3; Wilcoxon Test *p* = 0.0021, *p* = 0.068, and *p* = 0.54, respectively).

### Amount of images

Mean number of images acquired per training cycle was 2.83 ± 1.47. The number of images acquired per training cycle did not change significantly between the first and last training cycle (3.30 ± 2.23 and 2.73 ± 1.17, respectively, Wilcoxon signed-rank test *p* = 0.44).

### Subgroup analysis of ophthalmic assistants versus ophthalmologists

**Figure 4:**
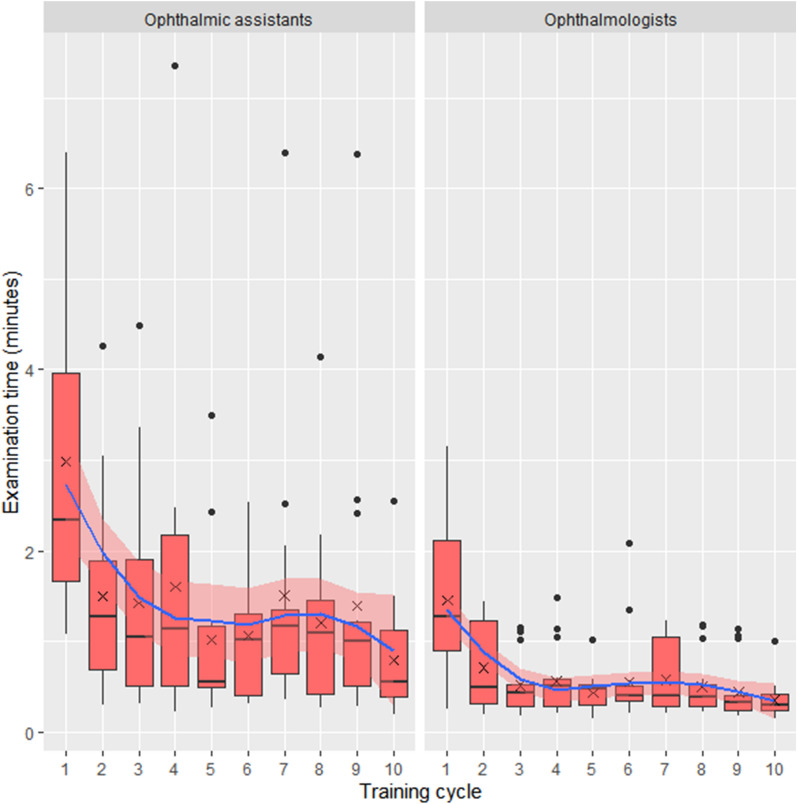
Effect of training on examination time-ophthalmic assistants versus ophthalmologists. Boxplot values over 1.5 interquartile range below the first quartile or above the third quartile were defined as outliers. Crosses indicate the mean. The blue line indicates a local polynomial regression fitting with 95% confidence intervals in light red.

Examination time significantly correlated with the number of training cycle for both ophthalmic assistants and ophthalmologists (Spearman correlation coefficient r = -0.37, *p* < 0.0001 and r = -0.31, *p* = 0.0003, respectively, see Fig. 4). Examination time was significantly shorter for ophthalmologists compared to ophthalmic assistants, at both the beginning and end of the training (1st training cycle: 1.45 ± 0.92 and 2.99 ± 1.72 min, Wilcoxon signed-rank *p* = 0.008; 10th cycle: 0.35 ± 0.21 and 0.79 ± 0.65 min, Wilcoxon signed-rank *p* = 0.011). Post-hoc analysis comparing differences between consecutive training cycles revealed significant differences only between the first and second training cycle.

There was no significant difference between ophthalmologists and ophthalmic assistants at the end of the training in usable field-of-view (96.8 ± 10.0% and 97.8 ± 4.74%, Wilcoxon signed-rank *p* = 0.78), sharpness/focus (Wilcoxon signed-rank *p* = 0.053), reflex artifacts (Wilcoxon signed-rank *p* = 0.076), and contrast/illumination (Wilcoxon signed-rank *p* = 0.083).

## Discussion

This study comprehensively analyzed SBFI learning curve dynamics and provided a comparison of expert and non-expert examiners. Our results emphasize the high accessibility and learnability of SBFI. The approximate half-hour SBFI training led to a significant improvement in examination time, usable field-of-view, and image quality. There was no significant difference between ophthalmologists and ophthalmic assistants except for examination time. The results of this study will aid implementing and planning further SBFI field studies.

The delegation of diagnostic tasks has already been proposed in the 1980’s for General Practitioners^34^ as well as in Ophthalmology, with medical assistants and non-medical personnel as the proposed staff for a variety of diagnostic measures including screenings for visual impairment, trachoma, glaucoma, and diabetic retinopathy^20,35–38^. Several studies on SBFI in clinical and outpatient settings have been conducted, with a variety of healthcare cadres performing the examinations^1–6,15,16,22–26^. This included ophthalmologists and optometrists familiar with ophthalmological diagnostics, but also nurses, technicians, other healthcare professionals, medical students and non-medical personnel without any prior experience in retinal imaging^3,24,26,39,40^. Whilst mastering direct ophthalmoscopy takes a lot of time and practice, the results of our study support the assumption that SBFI is fast to learn and easy to carry out for non-ophthalmologists^39^. Medical students, for example, learning both modalities achieved higher sensitivity and felt more comfortable when using SBFI^26,39^. Interestingly, the Smartphone Ophthalmoscopy Reliability Trial by Adam et al.^27^ found images were of higher quality when captured by an ophthalmology resident in contrast to medical students, while there was no statistically significant difference between Ophthalmologists and ophthalmic assistants except for examination time in our study. However, the results by Adam et al. were based on only two medical students and one ophthalmology resident. Queiroz et al. documented the rate of patients whose smartphone-based fundus images allowed clinical decision on daily basis over a 16 days period after an initial 4-h-training, however did not report any additional parameters, nor did perform any statistical analysis^41^. Still, their study supports that SBFI can be feasible for a low-cost diabetic retinopathy screening. Our study further supports these studies, as examiners with different medical backgrounds and levels of experience showed improvement in examination time, usable field-of-view, and image quality. Hence, SBFI might make the delegation of fundus imaging more feasible.

In fact, the first training cycle seems to be most relevant, as this was where most of the improvements occurred. Our results support existing data by Li et al. who compared SBFI examination time over a course of 4 training cycles using a model eye and found that most improvement occurred in the first training cycle^42^. Therefore, future SBFI trainings could potentially be shortened, however learning curve dynamics are most likely also dependent on the specific SBFI device used, compliance of the participants, and the employed health cadres.

As ophthalmologists are experienced with different fundus examination techniques, it is unsurprising that they were able to adapt more quickly to the SBFI diagnostic tool. Most ophthalmic assistants however are unexperienced with ophthalmoscopy. Nevertheless, the ophthalmic assistants included in our study learnt how to use the SBFI device quickly and produced good results which highlights the value of SBFI for delegation of fundus imaging tasks to non-ophthalmologists.

Image quality achieved was comparable to a previous study with this SBFI device^31^. Both studies used the same image quality scales for reflex artifacts and contrast/illumination, whereas the other study used an extended scale for sharpness/focus developed for direct comparison with conventional color fundus imaging^31^. While overall image quality was comparable, reflex artifacts seemed less prevalent in this study. The reason for this might be the much younger age of the participant sample and consequently the absence of pseudophakia. Pseudophakia is likely the main source of reflex artifacts for this SBFI device^31^.

Based on our results, one could argue to only use sharpness/focus as an image quality indicator in future field studies, as it was the only image quality parameter with significant improvement over the training course. However, this study included only 10 training cycles and a limited number of participants which is why other image quality parameters should not be discarded. Achieved image quality is likely not only depending on the examiner, but also on the patient sample and testing conditions (ambient light, possible need of protective equipment). Furthermore, reflex artifacts, contrast, and illumination are presumably influenced by lens status, fundus pigmentation and brightness of the adapter’s illumination^6^. Hence, all image quality parameters should be included in further field studies.

The strengths of our study are the prospective design and the comprehensive evaluation of SBFI learning curve dynamics for unexperienced users including examination time, usable field-of-view, image alignment, three parameters of image quality, and amount of acquired images. Furthermore, we compared ophthalmologists with ophthalmic assistants in a subgroup analysis. Limitations of our study are the small sample size, the exclusively young and healthy volunteer group with no opacification of any optical media or similarly challenging imaging conditions and the lack of different SBFI devices. Similar to the volunteers all examiners were young and had presumably a more intuitive understanding of handling SBFI devices compared to an older group of examiners who might have a lower smartphone affinity. However, this is purely speculative and has not been demonstrated yet. Another limitation is that the evaluation of usable field-of-view was carried out subjectively. All imaged eyes underwent pupillary dilation which-depending on the used SBFI adapter-may not be the case in the field and needs to be considered when extrapolating our findings to different settings.

In conclusion our study demonstrated that SBFI requires minimal training both for ophthalmologists and ophthalmic assistants, emphasizing its user-friendliness and its possibilities regarding task delegation and task shifting in low resource settings with few ophthalmologists. Additional studies are required to assess how our findings translate into a field study setting.
